# Non-Tuned Machine Learning Approach for Predicting the Compressive Strength of High-Performance Concrete

**DOI:** 10.3390/ma13051023

**Published:** 2020-02-25

**Authors:** Abobakr Khalil Al-Shamiri, Tian-Feng Yuan, Joong Hoon Kim

**Affiliations:** 1 Research Institute for Mega Construction, Korea University, Seoul 02841, Korea; abobakr2030@yahoo.com (A.K.A.-S.); yuantianfeng@korea.ac.kr (T.-F.Y.); 2 School of Civil, Environmental and Architectural Engineering, Korea University, Seoul 02841, Korea

**Keywords:** high-performance concrete, compressive strength, extreme learning machine, regularization, prediction

## Abstract

Compressive strength is considered as one of the most important parameters in concrete design. Time and cost can be reduced if the compressive strength of concrete is accurately estimated. In this paper, a new prediction model for compressive strength of high-performance concrete (HPC) was developed using a non-tuned machine learning technique, namely, a regularized extreme learning machine (RELM). The RELM prediction model was developed using a comprehensive dataset obtained from previously published studies. The input variables of the model include cement, blast furnace slag, fly ash, water, superplasticizer, coarse aggregate, fine aggregate, and age of specimens. *k*-fold cross-validation was used to assess the prediction reliability of the developed RELM model. The prediction results of the RELM model were evaluated using various error measures and compared with that of the standard extreme learning machine (ELM) and other methods presented in the literature. The findings of this research indicate that the compressive strength of HPC can be accurately estimated using the proposed RELM model.

## 1. Introduction

Concrete is the most commonly used structural material in the construction industry. It has several properties that make it more desirable than other construction materials. These properties include high strength, ease of fabrication, and high durability. Since different construction projects have specific performance requirements, improved concrete mixes known as high-performance concretes (HPCs) have been developed based on extensive research on concrete technology over the last three decades. The use of certain mineral and chemical admixtures such as fly ash and superplasticizer in HPC mixtures enhances the strength, durability, and workability of concrete. HPC is primarily used in bridges, tunnels, high-rise buildings, and hydropower structures.

The HPC mix design procedure requires several trial mixes to produce a concrete that meets the structural and environmental requirements of the construction project. This often results in a loss of time and materials. Compressive strength is one of the most important parameters in the design of HPC. It generally has a strong relationship with the overall quality of concrete. Early and accurate prediction of it can save time and cost by generating the required design data [1,2]. Conventional methods may not be suitable for predicting the compressive strength of HPC because the relationship between the concrete components and the compressive strength is highly nonlinear and, therefore, obtaining an accurate regression equation is difficult [3]. Several prediction models for compressive strength of different types of concrete have been developed using machine-learning (ML) techniques. These ML techniques include artificial neural network (ANN) [4,5,6,7,8,9], support vector machine (SVM) [10,11], and ensemble methods [12]. The compressive strength of fly ash concrete [13,14] and ground granulated blast furnace slag (GGBFS) concrete [15,16] was modeled using ANNs trained with a back-propagation (BP) algorithm. Cascardi et al. [17] used ANN to develop a prediction model for compressive strength of fiber reinforced polymer (FRP)-confined concrete. The developed model was formulated into a mathematical formula which could be useful for practical applications. Due to the environmental concerns regarding the scarcity of natural resources, several concrete mixtures have been designed with the use of recycled aggregates as replacement of natural materials. The influence of recycled aggregates, such as construction and demolition waste (CDW), on the compressive strength of concrete has been investigated using ANN in [18,19,20]. Yu et al. [21] proposed a novel approach based on SVM to predict the compressive strength of HPC. Behnood et al. [1] modeled the compressive strength of HPC using M5P model tree algorithm. Mousavi et al. [22] developed a gene expression programming (GEP)-based model for predicting the compressive strength of HPC. The proposed model outperformed the regression-based models. ANNs have gained more attention from ML researchers due to their universal approximation capability. Chithra et al. [23] investigated the applicability of ANN for predicting the compressive strength of HPC containing nanosilica and copper slag. Several other researchers have used ANN—either individually, as a hybrid with other methods, or in ensemble models to predict the compressive strength of HPC [3,12,24,25,26].

In the previous works, the modeling of concrete compressive strength was mostly carried out using classical neural networks trained with BP algorithm or other gradient-descent-based learning algorithms. These algorithms train all the parameters (i.e., weights and biases) of the network iteratively and may get stuck in local minima. Recently, a non-iterative learning method called extreme learning machine (ELM) has been proposed for training ANNs [27]. The output weights in ELM are analytically computed using the least-square method [28,29]. The hidden layer parameters (i.e., the input weights and hidden biases) are randomly assigned and need not be trained. These simplifications enable ELM to learn very quickly and achieve good generalization performance. However, since the standard ELM is based on the principle of empirical risk minimization, it may produce an overfitting model. Regularized extreme learning machine (RELM) [30] is an improved ELM method based on $L_{2}$ penalty (i.e., ridge regression), which provides better generalization performance than ELM. To the best of our knowledge, RELM has not been used for modeling the HPC strength.

The aim of this paper is to develop a new prediction model of compressive strength of HPC using the RELM method. The model was developed using 1133 experimental test results obtained from the literature. The prediction results of the developed RELM model were compared with that of the ELM and other individual and ensemble models reported in the literature. This investigation adds insights to the literature by highlighting the advantages of using ELM-based methods for predicting the compressive strength of concrete.

## 2. Experimental Dataset

**Figure 1:**
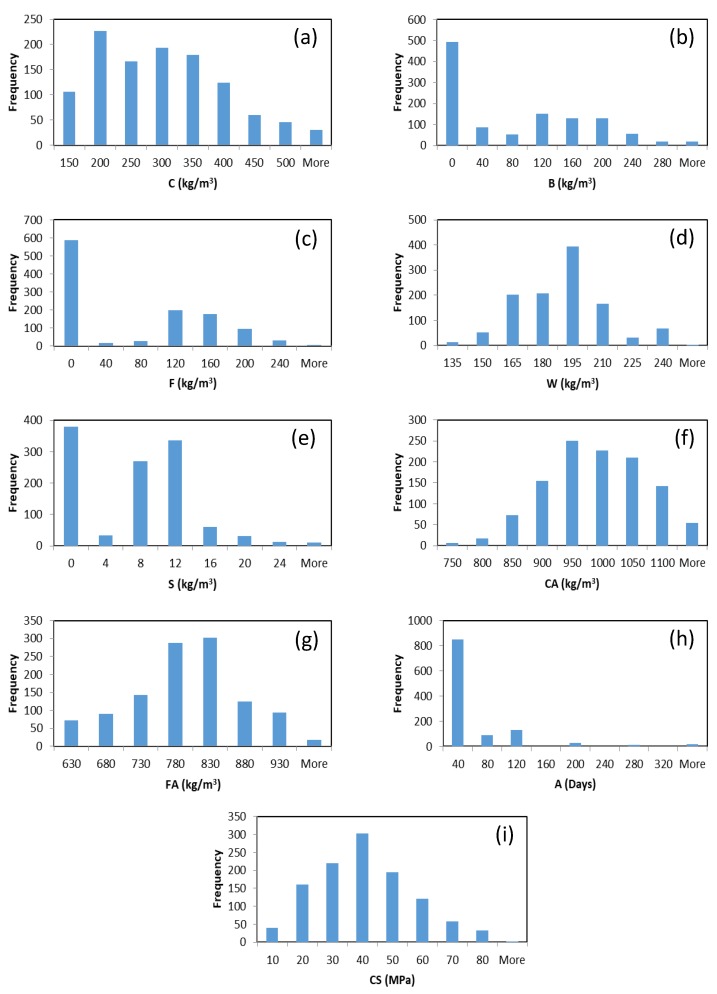
Histograms of the dataset variables.

A comprehensive dataset consisting of 1133 data records was obtained from the literature to develop the models [31,32]. This dataset has been used in many studies to develop prediction models for HPC strength [3,22,33]. The dataset contains eight input variables and one output variable. The input variables include cement (C), blast furnace slag (B), fly ash (F), water (W), superplasticizer (S), coarse aggregate (CA), fine aggregate (FA), and age of specimens (A). The output variable is the concrete’s compressive strength (CS). The compressive strength was calculated by uniaxial compressive strength test which was carried out according to ASTM C39. All the cylinders were made with ordinary Portland cement and cured under normal conditions. The statistical values of the dataset variables are shown in Table 1. Figure 1 shows the frequency histograms of the variables. For data interdependency analysis, the correlation coefficients between the predictor (i.e., input) variables were computed. As shown in Table 2, the values of the correlation coefficients indicate that there are no high correlations between the input variables. This is mainly due to the influence of high range of the data variables. In this research, the water to binder ratios were 24–90%, which almost include all concrete mixtures except ultra-high-performance concrete. In addition, two types of cementitious materials with a high range of replacement ratios (0–61%) were also considered.

## 3. Methods

### 3.1. Extreme Learning Machine

**Figure 2:**
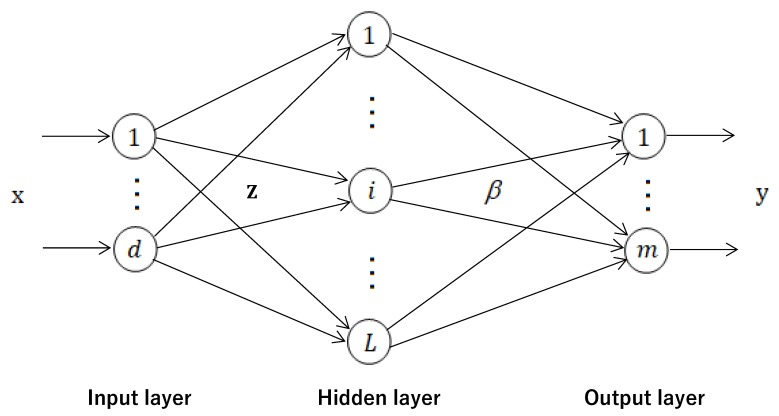
Architecture of the extreme learning machine (ELM).

Traditional algorithms for training ANN are usually based on a gradient descent approach in which the network weights and biases are tuned iteratively. Gradient-descent-based learning methods may get stuck in local minima or converge slowly. Huang et al. [27] proposed an efficient method for training ANN, called extreme learning machine (ELM). ELM significantly increases the speed of ANN learning process and obtains good generalization performance. In ELM, only the output weights of the network need to be determined (i.e., the hidden layer parameters are randomly initialized and fixed). No iterations are required for computing the output weights. The Moore–Penrose (MP) generalized inverse is used to determine the output weights [28,29,34]. Figure 2 shows a typical architecture of ELM with one hidden layer.

Consider *N* training samples ${\left(x_{i},t_{i}\right)}_{i=1}^{N}$, where $x_{i}=\left[x_{i1},x_{i2},\ldots ,x_{id}\right]\in R^{d}$ and $t_{i}=\left[t_{i1},t_{i2},\ldots ,t_{im}\right]\in R^{m}$. Let *L* denote the number of neurons in the hidden layer of an ANN. If this ANN with random hidden neurons can approximate these *N* training examples with zero error, the output of ANN will be as follows:

(1) $$f\left(x_{j}\right)=\sum_{i=1}^{L}\beta_{i}h_{i}\left(x_{j}\right)=h\left(x_{j}\right)\beta =t_{j},\;\;j=1,\ldots ,N,$$

where $\beta_{i}=\left[\beta_{i1},\beta_{i2},\ldots ,\beta_{im}\right]$ is the weight vector connecting the *i*th hidden neuron to *m* output neurons, $h_{i}\left(x_{j}\right)=a\left(z_{i},b_{i},x_{j}\right)$ is the output of the *i*th neuron in the hidden layer, where $z_{i}\in R^{d}$ and $b_{i}\in R$ are the input weights and bias of the *i*th hidden neuron, respectively. a(·) is the hidden neuron activation function which can be a sigmoid, Gaussian, or any function satisfying the universal approximation capability theorems of ELM [29,35,36]. $h\left(x_{j}\right)=\left[h_{1}\left(x_{j}\right),h_{2}\left(x_{j}\right),\ldots ,h_{L}\left(x_{j}\right)\right]$ is the hidden layer output vector corresponding to the input $x_{j}$. $\beta ={\left[\beta_{1},\beta_{2},\ldots ,\beta_{L}\right]}^{T}$ is the output weight matrix. Equation (1) can be written compactly as follows [28]:

(2) Hβ=T,

where H is the hidden layer output matrix of ELM [37]:

(3) $$H=\left[\begin{array}{c} h(x_{1})\\ \vdots \\ h(x_{N}) \end{array}\right]=\left[\begin{array}{ccc} a(z_{1},b_{1},x_{1}) & \ldots & a(z_{L},b_{L},x_{1})\\ \vdots & \ldots & \vdots \\ a(z_{1},b_{1},x_{N}) & \ldots & a(z_{L},b_{L},x_{N}) \end{array}\right],$$

and **T** is the target matrix of the training data:

(4) $$T=\left[\begin{array}{c} t_{1}\\ \vdots \\ t_{N} \end{array}\right]=\left[\begin{array}{ccc} t_{11} & \ldots & t_{1m}\\ \vdots & \ldots & \vdots \\ t_{N1} & \ldots & t_{Nm} \end{array}\right].$$

The parameter β can be computed as follows [27]:

(5) $$\beta =H^{†}T,$$

where $H^{†}$ is the MP generalized inverse of **H** [38], which can be computed using different methods such as orthogonal projection method and singular value decomposition (SVD) [39]. If $HH^{T}$ is nonsingular, the orthogonal projection method computes $H^{†}$ as $H^{T}{\left(HH^{T}\right)}^{-1}$; otherwise, $H^{†}$ = ${\left(H^{T}H\right)}^{-1}H^{T}$ when $H^{T}H$ is nonsingular [40].

### 3.2. Regularized Extreme Learning Machine

Even though the standard ELM is designed to provide good generalization performance at fast learning speed, it may tend to produce an overfitting model because it is based on the empirical risk minimization (ERM) principle [30,41,42]. The ELM solution may not be stable if the hidden layer output matrix H is an ill-conditioned matrix. To overcome these problems, regularization is used in ELM [30]. Based on ridge regression theory [43], if a positive value is added to the diagonal of $HH^{T}$ or $H^{T}H$, the solution of ELM will be more stable and provide better generalization performance [30,40]. Therefore, the solution (i.e., the output weights β) of the RELM method can be calculated as follows [30]: if the number of hidden neurons is less than the number of training examples, then

(6) $$\beta ={\left(\frac{I}{\lambda }+H^{T}H\right)}^{-1}H^{T}T;$$

otherwise,

(7) $$\beta =H^{T}{\left(\frac{I}{\lambda }+HH^{T}\right)}^{-1}T,$$

where I is an identity matrix and λ is the regularization parameter. The steps of the RELM method are given in Algorithm 1 [30].
**Algorithm 1:** Regularized extreme learning machine (RELM) Algorithm
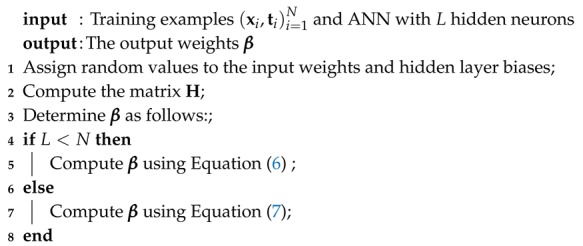


## 4. Experimental Setting

**Figure 3:**
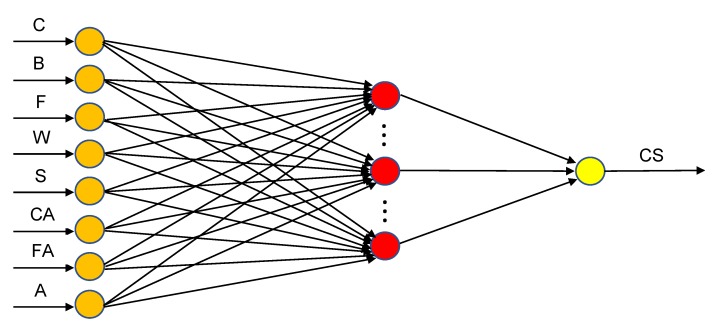
The network architecture used in the ELM and RELM models.

The network architecture used in this paper was a feedforward network with a single hidden layer. As shown in Figure 3, the compressive strength of HPC is represented by one neuron in the output layer. The input layer of the network contains eight neurons which represent the input variables: C, B, F, W, S, CA, FA, and A. Sigmoid function a(x)=1/(1+exp(−x)) was used as an activation function in the hidden layer. According to the ELM theory, a good generalization performance can be obtained if the number of neurons in the hidden layer is large enough [28,40,44]. This is due to the random determination of the hidden layer parameters. The number of hidden neurons was selected from the range [50,60,…,300]. To find the optimal number of hidden neurons, each network architecture was evaluated based on cross-validation method. For ELM, the optimal number of hidden neurons was 230. RELM is not very sensitive to the size of the hidden layer, provided that the number of hidden neurons is large enough and the parameter λ is appropriately chosen [40]. For RELM, similar to [40], the number of hidden neurons was set to 1000 and the parameter λ was chosen from the range $\left[2^{5},2^{20}\right]$. The input variables were normalized into the range of [LB,UB] using the following equation:

(8) $$X_{n}=pX_{o}+q,$$

where

(9) $$p=\frac{UB-LB}{X_{max}-X_{min}}$$

and

(10) $$q=LB-pX_{min},$$

in which $X_{n}$ and $X_{o}$ are the normalized and original values of the input variable, respectively. $X_{max}$ and $X_{min}$ are the maximum and minimum values of the corresponding input variable, respectively. In this paper, LB=−1 and UB=1.

### Performance-Evaluation Measures and Cross Validation

In this paper, the prediction accuracy of the ELM and RELM models was evaluated using root mean squared error (RMSE), mean absolute error (MAE), mean absolute percentage error (MAPE), and the Pearson correlation coefficient (R). These statistical measures are widely used in the literature and are expressed as follows:

(11) $$RMSE=\sqrt{\frac{1}{n}\sum_{i=1}^{n}{\left(t-y\right)}^{2}},$$

(12) $$MAE=\frac{1}{n}\sum_{i=1}^{n}|t-y|,$$

(13) $$MAPE=\frac{1}{n}\sum_{i=1}^{n}\left|\frac{t-y}{t}\right|,$$

(14) $$R=\frac{\sum_{i=1}^{n}\left(t-\bar{t}\right)\left(y-\bar{y}\right)}{\sqrt{\sum_{i=1}^{n}{\left(t-\bar{t}\right)}^{2}\sum_{i=1}^{n}{\left(y-\bar{y}\right)}^{2}}},$$

where *t* and *y* are the experimental and predicted values of compressive strength, respectively. *n* is the number of data instances, $\bar{t}$ is the mean of the experimental values of compressive strength, and $\bar{y}$ is the mean of the predicted values of compressive strength.

**Figure 4:**
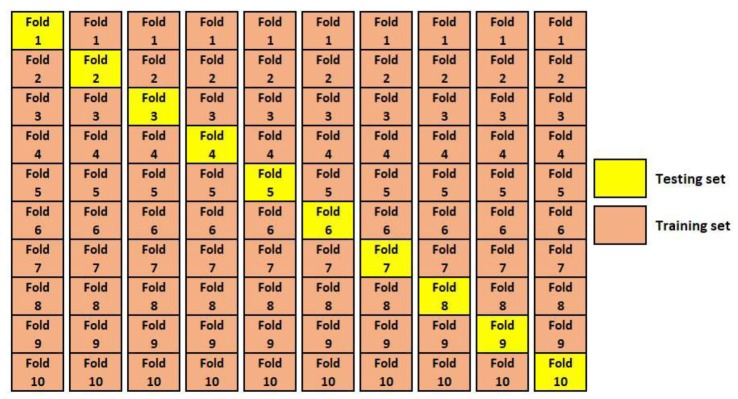
The ten-fold cross-validation method.

The *k*-fold cross-validation procedure is often used to minimize bias associated with random division of the dataset into training and testing sets. In *k*-fold cross-validation, the dataset is divided into *k* partitions (e.g., k=5 or k=10). Each partition of the data is called a fold. A single fold is used to test the model and the remaining k−1 folds are used to train the model. This process is repeated *k* times, each time with a different testing set. After running cross-validation, the mean and standard deviation of the performance measures are computed. The ten-fold cross-validation method is shown in Figure 4. In this paper, ten-fold cross-validation was used to assess the prediction capability of the ELM and RELM models.

**Figure 5:**
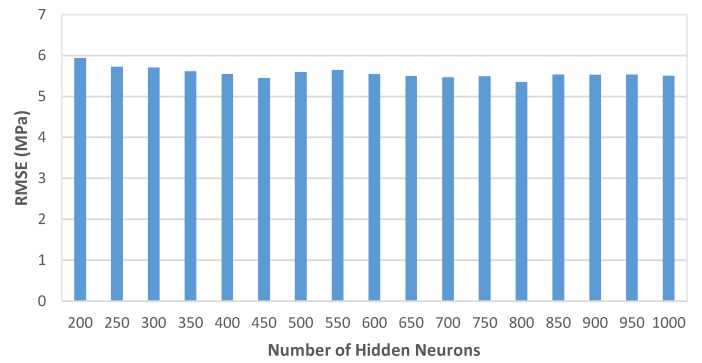
Average root mean squared error (RMSE) values of the RELM method with different network architectures.

**Figure 6:**
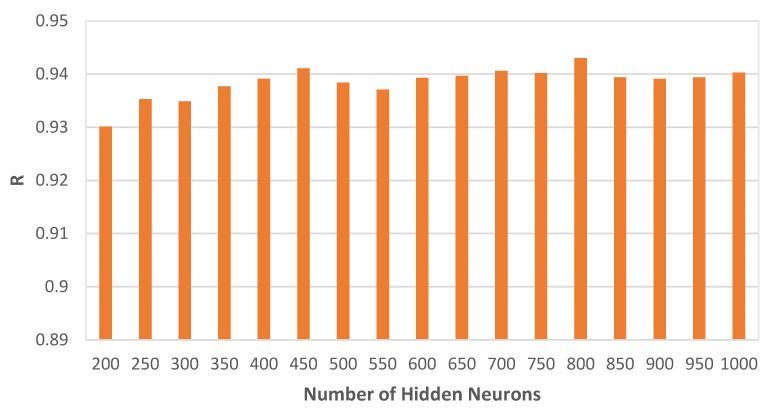
Average Pearson correlation coefficient (R) values of the RELM method with different network architectures.

In this paper, as it has been mentioned above, the number of hidden neurons for the RELM model was set to 1000. To see how the RELM method performs with a varying number of neurons, several experiments were conducted and the results are shown in Figure 5 and Figure 6. It can be observed that the RELM method is stable, not very sensitive to the number of hidden neurons, and good predictions can be obtained.

## 5. Results and Discussion

The prediction results—in terms of the average values of different statistical measures—of the ELM and RELM models are shown in Table 3. From Table 3, it can be observed that the developed RELM model achieves better performance than the ELM model in all the statistical measures on the training set. It obtains 3.6737 and 0.9736 in the RMSE and R measures, respectively. The corresponding values obtained by the ELM model are 4.1846 and 0.9656. The good results obtained by the RELM model on the training set indicate the predictive capability of the developed model. For testing set, the RELM model outperforms the ELM model by obtaining the lowest values in the RMSE, MAE, and MAPE error measures and the highest value in the R measure. The obtained R-value of the RELM model on the testing set is 0.9403, which indicates that there is a strong correlation between the experimental and predicted values of the compressive strength. The accurate predictions of the developed RELM model on the testing set suggest that the model is able to generalize well to unseen data.

Table 4 shows the standard deviations of the RMSE measure for the ELM and RELM models. The standard deviations for the RELM model on the training, testing, and all data sets are 0.0405, 0.5054, and 0.0771, respectively, which are lower than that for the ELM model. From Table 3 and Table 4, it can be observed that the developed RELM model not only achieves accurate predictions on average, but also obtains low standard deviations, which supports the reliability of the RELM model for predicting the HPC compressive strength.

The prediction results of the ELM and RELM models were also compared with that of the individual and ensemble methods presented in [3]. The individual methods include ANN trained by BP algorithm, classification and regression trees (CART), Chi-squared automatic interaction detection (CHAID) technique, linear regression (LR), generalized linear model (GENLIN), and SVM. A brief introduction to these techniques is presented in [3]. The ensemble methods were modeled by combining the best-performing individual models [3].

Table 5 shows that the ANN model has the best performance among the individual methods reported in [3]. The values of the RMSE, MAE, and MAPE measures for ANN are 6.329, 4.421, and 15.3, respectively, which are the lowest compared to that for the other five individual methods in [3]. However, the ELM model outperforms ANN in the RMSE and MAPE measures and obtains comparable performance in the correlation coefficient measure. It obtains 6.0377 and 15.2558 in the RMSE and MAPE measures, respectively. It can be seen that the ELM model outperforms SVM, which is the second-best individual model in [3], in all the error measures. As shown in Table 5, the combination of the individual ANN and SVM methods yielded the best ensemble model among the ensemble methods. The ELM model obtains better performance than the ensemble ANN+SVM method only in the RMSE measure. From Table 5, it can be observed that the proposed RELM model has the best performance compared to the ELM model and the other individual and ensemble methods in all the performance measures. The high predictive accuracy of the RELM model suggests that the model developed is a reliable method for estimating the compressive strength of HPC.

**Figure 7:**
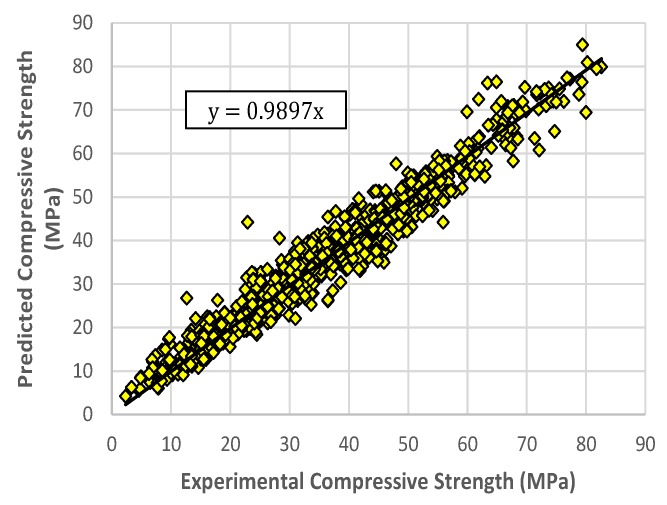
Predicted versus experimental compressive strength values, RELM model for training data.

**Figure 8:**
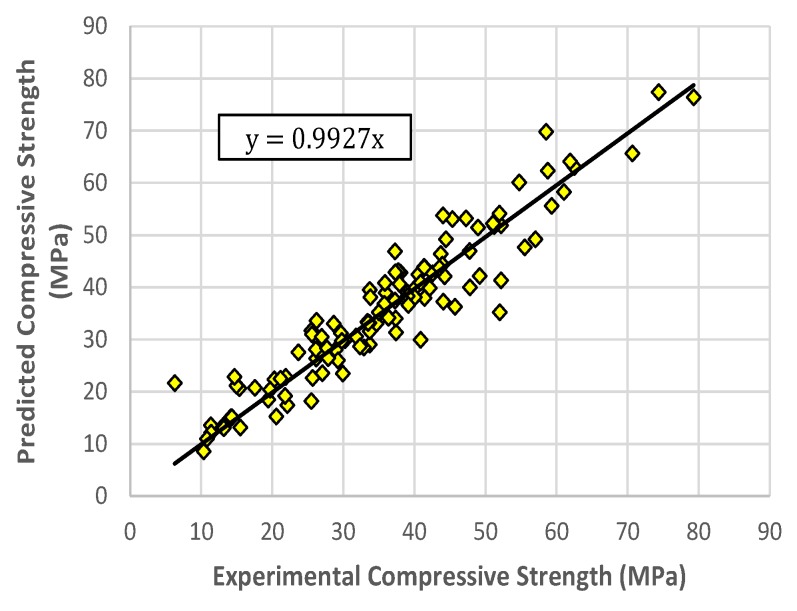
Predicted versus experimental compressive strength values, RELM model for testing data.

The values in Table 3 represent the average performance of the models. The representative RELM model was selected based on its performance in the RMSE measure on the testing and on all data sets. The selected RELM model obtained 3.6789 4.7459, and 3.7998 in the RMSE measure on the training, testing, and all data sets, respectively. The corresponding R-values are 0.9741, 0.9459, and 0.9717. The experimental values of compressive strength versus the predicted ones using the RELM model for the training and testing sets are shown in Figure 7 and Figure 8, respectively. It can be observed that the points are distributed close to the regression lines, with the values of the slopes for training and testing sets of 0.9897 and 0.9927, respectively. This indicates good agreement between the experimental values and the predicted values obtained by the RELM model.

**Figure 9:**
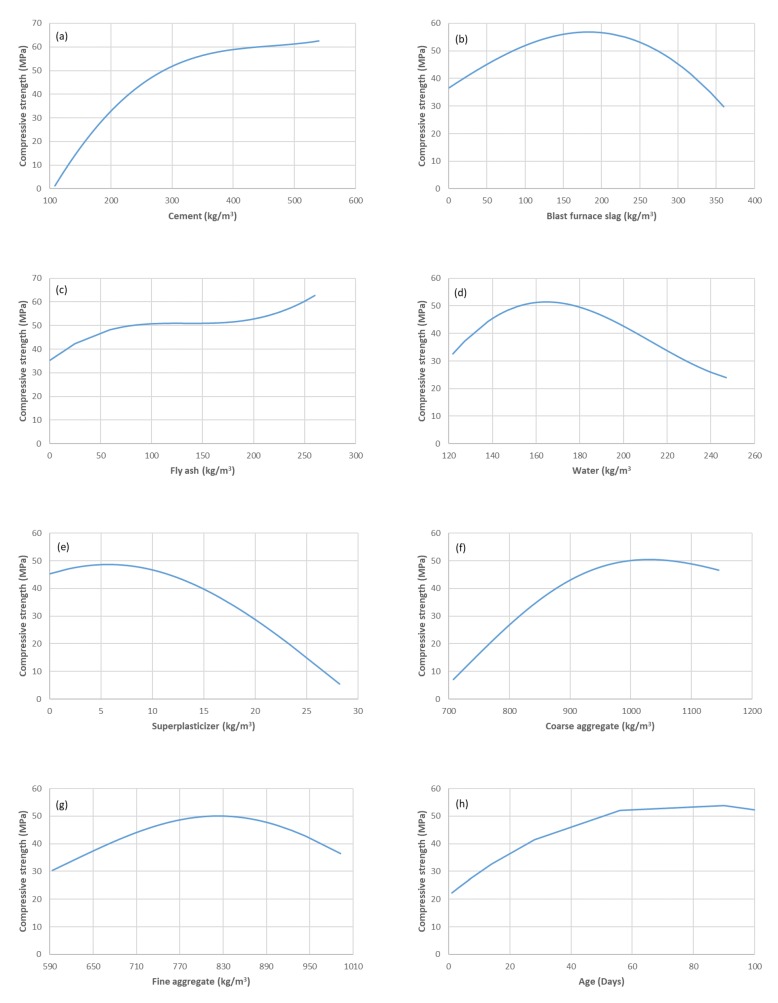
Sensitivity analysis of the developed RELM model.

A sensitivity analysis was performed to investigate the response of the developed RELM model to the changes of the input variables. In the analysis, only one input variable was changed at a time and the remaining input variables were kept constant at their average values [25,33]. The results of the sensitivity analysis using the RELM model are shown in Figure 9. It can be observed that the results of the analysis indicate well-known properties of HPC that have been described in several published papers in the literature. For example, in Figure 9a, the quantity of cement has a direct influence on hydration degree, and the degree of cement hydration has a direct effect on porosity and consequently on strength. This is because of the pore refinement associated with the pozzolanic reaction and the increase in Calcium-Silicate-Hydrate (C-S-H).

In general, the models developed using ML techniques or similar approaches are valid only for the range of data used for their development. However, it is recommended to consider the range of data variables presented in Table 1 when using the developed RELM model to compute the concrete compressive strength.

## 6. Conclusions

In the construction industry, developing a prediction model that provides accurate and early estimation of compressive strength of concretes is very important as it can help in saving time and costs by providing the required design data. In this paper, a regularized ELM model (RELM) was developed, using a comprehensive database obtained from previous works, for estimating the compressive strength of HPC. The findings of this research are outlined as follows:

- Although the ELM model achieves good generalization performance (R = 0.929 on average), the RELM model performs even better.
- This research confirms that the use of regularization in ELM could prevent overfitting and improve the accuracy in estimating the HPC compressive strength.
- The RELM model can estimate the HPC compressive strength with higher accuracy than the ensemble methods presented in the literature.
- The proposed RELM model is simple, easy to implement, and has a strong potential for accurate estimation of HPC compressive strength.
- This work provides insights into the advantages of using ELM-based methods for predicting the compressive strength of concrete.
- The prediction performance of the ELM-based models can be improved by optimizing the initial input weights using optimization techniques such as harmony search, differential evolution, or other evolutionary methods.

## Figures and Tables

**Table 1:** The statistical values of the dataset variables.

Variable	Minimum	Maximum	Average	Standard Deviation
C (kg/m${}^{3}$)	102.00	540.00	276.51	103.47
B (kg/m${}^{3}$)	0.00	359.40	74.27	84.25
F (kg/m${}^{3}$)	0.00	260.00	62.81	71.58
W (kg/m${}^{3}$)	121.80	247.00	182.99	21.71
S (kg/m${}^{3}$)	0.00	32.20	6.42	5.80
CA (kg/m${}^{3}$)	708.00	1145.00	964.83	82.79
FA (kg/m${}^{3}$)	594.00	992.60	770.49	79.37
A (Days)	1.00	365.00	44.06	60.44
CS (MPa)	2.33	82.60	35.84	16.10

**Table 2:** Correlation coefficients between the input variables.

Variable	C	B	F	W	S	CA	FA	A
C	1.0000	−0.2728	−0.4204	−0.0890	0.0674	−0.0730	−0.1859	0.0906
B	−0.2728	1.0000	−0.2889	0.0995	0.0527	−0.2681	−0.2760	−0.0442
F	−0.4204	−0.2889	1.0000	−0.1508	0.3528	−0.1055	−0.0062	−0.1631
W	−0.0890	0.0995	−0.1508	1.0000	−0.5882	−0.2708	−0.4247	0.2420
S	0.0674	0.0527	0.3528	−0.5882	1.0000	−0.2747	0.1985	−0.1984
CA	−0.0730	−0.2681	−0.1055	−0.2708	−0.2747	1.0000	−0.1534	0.0233
FA	−0.1859	−0.2760	−0.0062	−0.4247	0.1985	−0.1534	1.0000	−0.1394
A	0.0906	−0.0442	−0.1631	0.2420	−0.1984	0.0233	−0.1394	1.0000

**Table 3:** Prediction results of the ELM and RELM models.

Model	Dataset	RMSE (MPa)	MAE (MPa)	MAPE (%)	R
ELM	Training data	4.1846	3.2062	11.3922	0.9656
	Testing data	6.0377	4.4419	15.2558	0.929
	All data	4.4087	3.3298	11.7787	0.9617
RELM	Training data	3.6737	2.7356	9.74	0.9736
	Testing data	5.5075	3.9745	13.467	0.9403
	All data	3.8984	2.8595	10.1125	0.9702

**Table 4:** The standard deviations of the RMSE measure for the ELM and RELM models.

Model	Training Data	Testing Data	All Data
ELM	0.1001	0.6739	0.1401
RELM	0.0405	0.5054	0.0771

**Table 5:** Generalization performance comparison of ELM, RELM, and other methods presented in [3].

Method	Testing Data			
	RMSE (MPa)	MAE (MPa)	MAPE (%)	R
ELM	6.0377	4.4419	15.2558	0.929
RELM	5.5075	3.9745	13.467	0.9403
Individual methods [3]:				
ANN	6.329	4.421	15.3	0.930
CART	9.703	6.815	24.1	0.840
CHAID	8.983	6.088	20.7	0.861
LR	11.243	7.867	29.9	0.779
GENLIN	11.375	7.867	29.9	0.779
SVM	6.911	4.764	17.3	0.923
Ensemble methods [3]:				
ANN + CHAID	7.028	4.668	16.2	0.922
ANN + SVM	6.174	4.236	15.2	0.939
CHAID + SVM	6.692	4.580	16.3	0.929
ANN + SVM + CHAID	6.231	4.279	15.2	0.939
